# The evolutionary conservation of eukaryotic membrane-bound adenylyl cyclase isoforms

**DOI:** 10.3389/fphar.2022.1009797

**Published:** 2022-09-27

**Authors:** Joachim E. Schultz

**Affiliations:** Department Pharmaceutical Biochemistry, Pharmazeutisches Institut der Universität, Tübingen, Germany

**Keywords:** adenylyl cyclase, isozyme, domain structure, evolution, bioinformatics, regulation, cyclic AMP, conservation

## Abstract

The nine membrane-delimited eukaryotic adenylyl cyclases are pseudoheterodimers with an identical domain order of seven (nine) distinct subdomains. Bioinformatics show that the protein evolved from a monomeric bacterial progenitor by gene duplication and fusion probably in a primordial eukaryotic cell around 1.5 billion years ago. Over a timespan of about 1 billion years, the first fusion product diverged into nine highly distinct pseudoheterodimeric isoforms. The evolutionary diversification ended approximately 0.5 billion years ago because the present isoforms are found in the living fossil coelacanth, a fish. Except for the two catalytic domains, C1 and C2, the mAC isoforms are fully diverged. Yet, within each isoform a high extent of conservation of respective subdomains is found. This applies to the C- and N-termini, a long linker region between the protein halves (C1b), two short cyclase-transducing-elements (CTE) and notably to the two hexahelical membrane domains TM1 and TM2. Except for the membrane anchor all subdomains were previously implicated in regulatory modalities. The bioinformatic results unequivocally indicate that the membrane anchors must possess an important regulatory function specifically tailored for each mAC isoform.

## Introduction

Isozymes are ubiquitous throughout all kingdoms of life. They either are splice variants of a “master” gene or are encoded by separate genes. Often, they share stretches of conserved amino acid sequences pointing to gene duplication events during evolution followed by subsequent divergence restricted by functional requirements. Frequently, this results in the evolution of new regulatory features in individual isoforms whereas the functional catalytic center is preserved. A textbook example is hexokinase 1 on chromosome 10 and glucokinase on chromosome 7 (human), both catalyzing glucose phosphorylation generating glucose-6-phosphate. Hexokinase is subject to product inhibition and glucokinase is not. Expression of these isozymes is tissue specific. Thus, regulation differs in two important aspects, different kinetics and cellular localization. Numerous other examples show similar patterns, i.e., identical reaction yet different regulation.

A most interesting family of multiple isozymes are class III adenylyl cyclases (AC) which are present in pro-as well as in eukaryotes. In the latter, only class IIIa isozymes are present whereas in bacteria classes IIIa to IIId forms exist (Linder and Schultz, 2003). These ACs catalyze the cyclization of ATP to the universal second messenger cyclic 3′, 5′-AMP by an essentially identical reaction mechanism in an endothermic reaction driven by the hydrolysis of the product pyrophosphate (Hayaishi et al., 1971). In all instances, the active centers are dimeric.

**FIGURE 1:**
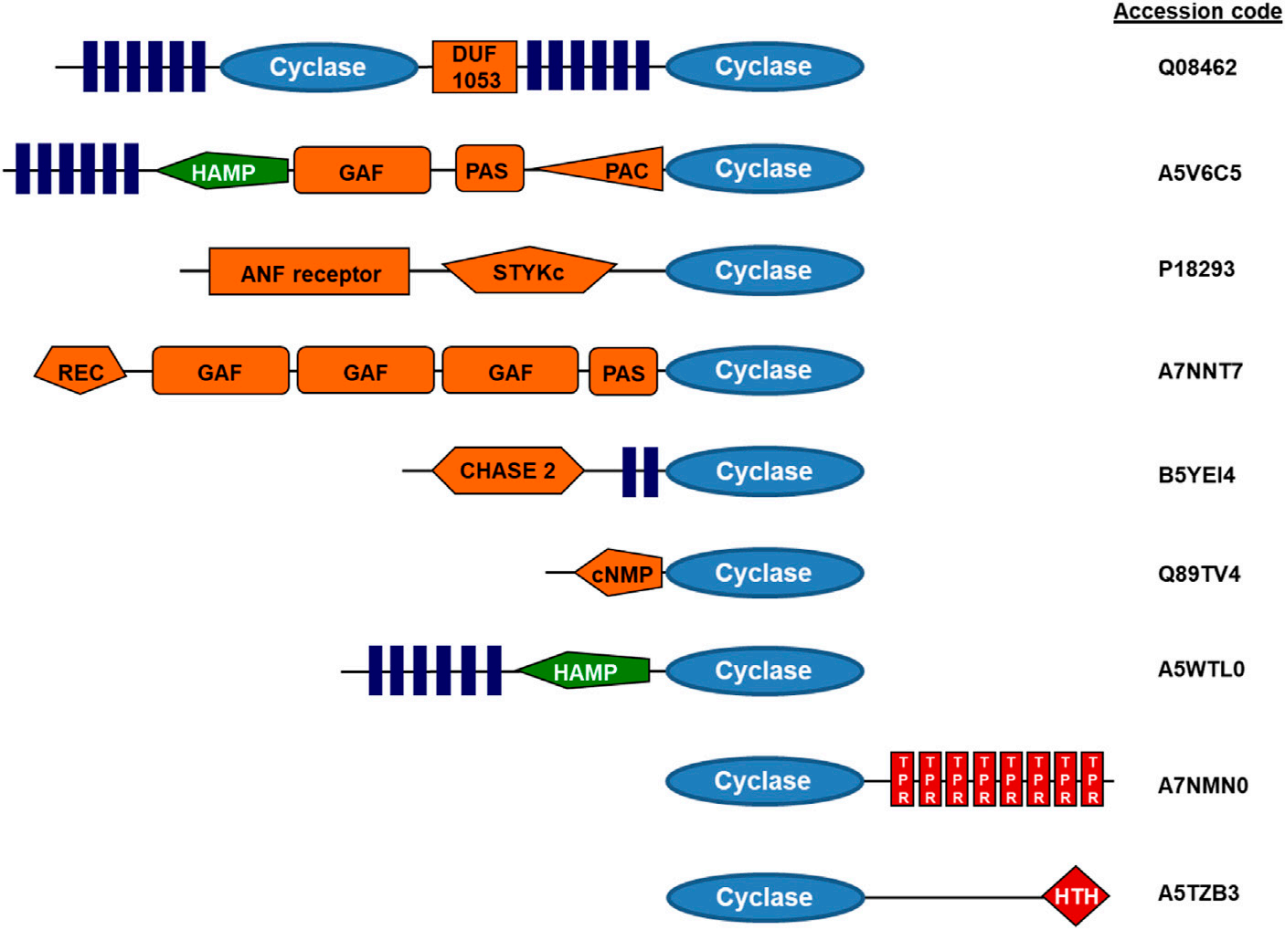
Examples of modular adenylyl cyclase proteins (domain symbols adapted from EMBL-SMART). Q08462, human adenylyl cyclase, type 2; A5V6C5, adenylyl cyclase from Sphingomonas wittichii; A7NNT7, Roseiflexus castenholzi adenylyl cyclase with GAF and PAS/PAC sensors; B5YEI4, Dictyoglomus thermophilum adenylyl cyclase; Q89TV4, Bradyrhizobium japonicum adenylyl cyclase; A5WTL0, mycobacterial adenylyl cyclase Rv3645; A7NMN0, Roseiflexus castenholzii adenylyl cyclase with terminal TPR repeats; A5TZB3, mycobacterial adenylyl cyclase Rv0386 with a helix-turn-helix terminal transcriptional regulator (figure adapted from ref. Schultz and Natarajan, 2013).

Bacterial class III ACs are monomeric proteins, which homodimerize to form a catalytic center at the dimer interface. It is unknown whether this is of regulatory importance. In individual bacterial strains up to 30 class III AC isozymes have been identified, all containing highly similar catalytic domains (https://www.ncbi.nlm.nih.gov/Complete_Genomes/SignalCensus.html) (Bassler et al., 2018). A different picture emerges regarding associated domains which are not required for activity. In bacterial ACs numerous diverse N-terminal domains have been identified, among them membrane anchors of two, four or six predicted α-helices, and several distinct domains located between membrane anchor and catalytic domain (Linder and Schultz, 2003; Schultz and Natarajan, 2013; Beltz et al., 2016; Bassler et al., 2018; Wissig et al., 2019). Thus, each of these bacterial AC isoforms probably is endowed with unique molecular features, which confer peculiar regulatory modalities, almost completely unexplored at this time (for representative samples see Figure 1 and (Bassler et al., 2018)). Another interesting question is why many bacteria contain multiple class III AC isozymes, e.g., 16 in *Mycobacterium tuberculosis* or 28 in *Sinorhizobium meliloti*. Notably, the AC CyaC from *Sinorhizobium* is regulated by its hexahelical membrane domain which contains a di-heme-B entity integrated in its membrane domain enabling regulation by oxidation-reduction processes (Wissig et al., 2019). The regulation of other bacterial ACs with hexahelical membrane domains is unknown. In general, very little is known about how the expression of multiple class III ACs in bacteria is coordinated. It is reasonable to assume that bacteria use the regulatory diversity of ACs to specifically respond to biochemical or biophysical cues when encountering variant environments. This area deserves further study in the future.

In contrast, the regulation of the nine membrane-delimited vertebrate AC isoforms (mACs) has been explored extensively, has often been reviewed over the years and this shall not be recapitulated here (Sinha and Sprang, 2006; Dessauer et al., 2017; Ostrom et al., 2022). The current canonical view is that mammalian mACs are indirectly regulated via GPCR-receptor stimulation and subsequent cytosolic release of and activation by the Gsα subunit of the trimeric G-proteins. Other regions have been implicated to different extents such as the N-and C-termini, and the long C1b loop connecting both halves of mAC proteins. In contrast, the extensive AC membrane anchors usually have not been implicated in direct regulatory processes of eukaryotic mACs. Since 2010, evidence emerged in our laboratory that the membrane domains might directly affect regulation of vertebrate mACs, i.e., may operate as receptors for yet unknown ligands (Kanchan et al., 2010; Winkler et al., 2012; Beltz et al., 2016; Seth et al., 2020).

Here, I present very extensive comparisons of eukaryotic mAC protein sequences, either comprising mACs of all nine AC isoforms together or of individual isoforms alone. Bioinformatics indicate that all domains, N-terminus, both hexahelical membrane anchor domains and C-terminal domains most likely have isoform-specific physiological roles which are distinct for every mAC isoform and subdomain. The existence of conserved cyclase-transducing elements (CTEs) between the exits of the two TM domains and the adjoining catalytic C1 and C2 domains strongly argues for a regulatory input *via* the membrane domains. This evidence is derived from evolutionary and genetic considerations, from sequence and functional comparisons, from structural work and from our own ongoing experimental work. In this perspective these data are compiled and presented in easily comprehensible diagrams in which individual aa are hidden by respective shading.

## The evolution of pseudoheterodimeric adenylyl cyclases

**FIGURE 2:**
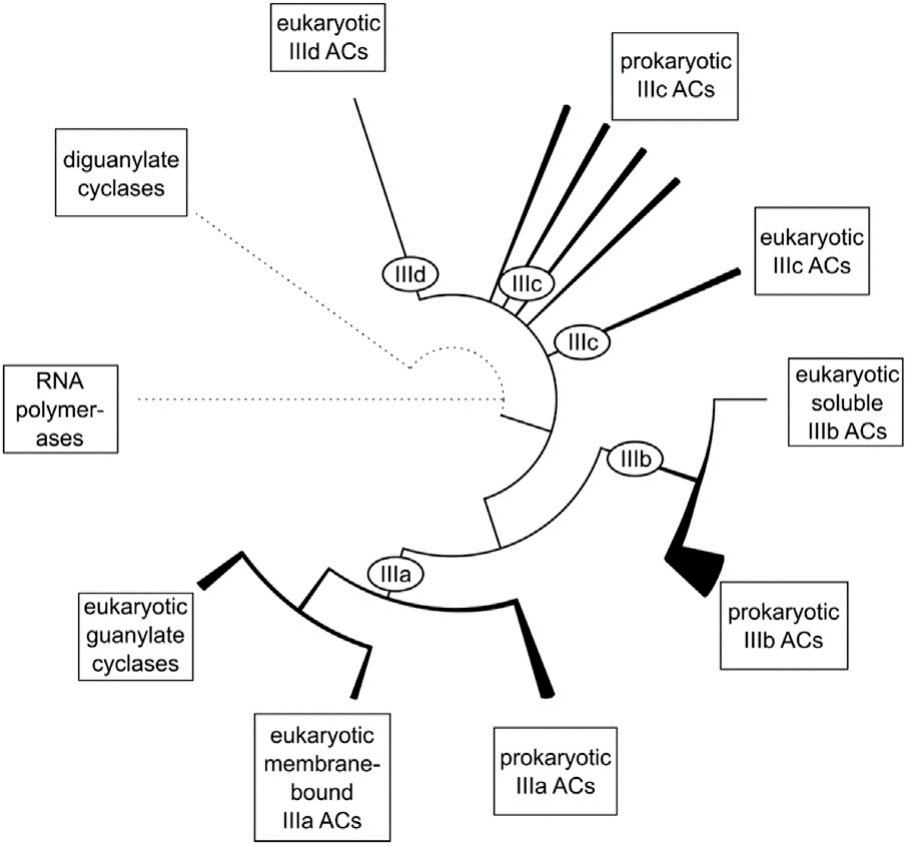
The evolutionary relationship between the catalytic domains of class III adenylyl cyclases. Dotted lines represent remote homology to other protein families. Solid lines represent relations between major subgroups of class III ACs. Within class III ACs, the line thickness symbolizes the diversity of domain architectures within a branch. Subclasses IIIc and IIId consist of several groups of equal rank and might be collectively referred to as subclass IIIc/d (figure from ref. Bassler et al., 2018).

Bacterial and eukaryotic class III ACs including eukaryotic guanylyl cyclases have a common evolutionary root as reported earlier [Figure 2; (Bassler et al., 2018)]. Obviously, the eukaryotic mAC isozymes are the result of an early gene duplication and subsequent fusion event of one of the bacterial progenitor ACs. Many bacterial ACs have a single hexahelical membrane domain and an inactive catalytic domain which requires complementation by dimerization for enzymatic activity as exemplified by the mycobacterial AC Rv1625c (Guo et al., 2001). Both bacterial monomers participate equally in forming two productive active centers (Guo et al., 2001; Reddy et al., 2001; Sinha et al., 2005; TewsFindeisen et al., 2005). The first gene-duplication-fusion event most likely occurred early after the emergence of eukaryotic cells around 1.5 billion years ago resulting in a linked homodimeric protein (Farquhar et al., 2007; Bobrovskiy et al., 2018; Tang et al., 2020). Subsequent gene duplications concomitant with mutational diversification finally resulted in nine distinct and, importantly, functionally indispensable eukaryotic mAC isoforms. This process appears to have ended rather abruptly after about one billion years of evolution, i.e., around 0.5 billion years ago. Around this time the nine mAC isoforms as we know them today had evolved in the coelacanth and elephant shark. Interestingly, in these fish also soluble and membrane-bound guanylyl cyclases were identified indicating that at this point of evolution, i.e., 0.5 billion years ago, the separation of ACs and GCs was probably evolutionarily established. Coelacanth and elephant shark have a stable 0.5-billion-year evolutionary history and the last eukaryotic common ancestor these fish shared with humans was alive about 450 million years ago. For example, the mAC5 isoforms from human and coelacanth, and elephant shark, share overall 66% and 59% sequence identity, respectively. These considerations bolster the claim that at this point in evolution each isoform had acquired molecular features which were functionally indispensable for regulation of eukaryotic cells and organisms. Any subsequent mutational diversification obviously did not result in an added evolutionary advantage, rather, we can suppose, was detrimental. It appears then fair to state that each mAC isoform appears to be exceedingly well conserved across all species independently of their evolutionary position. Probably in this aeon also GPCRs and G-proteins evolved which are absent in archaea and eubacteria (Noonan et al., 2004; Anantharaman et al., 2011; Amemiya et al., 2013; Bradford et al., 2013).

In bacteria, a fused dimeric class III AC species is unknown. An open question is what is the advantage of a duplicated and fused AC protein compared to the bacterial monomer-dimer equilibrium? Conceivably, in a linked dimer the number and specificity of regulatory inputs could be considerably expanded (see below). Further, a physically linked dimer might respond to regulatory inputs more readily and reversibly.

## Similarity of adenylyl cyclase isoforms

The genes of the human mAC isozymes are distributed over eight chromosomes, AC1 is on chromosome 7, AC2 on 5, AC3 on 2, AC4 on 14, AC5 on 3, AC6 on 12, AC7 on 16, AC8 on 8, and AC9 on 16. Probably, this suggests specific expression patterns, cellular localization, tissue distribution and specialized functions. No detailed studies are available on how expression of mAC isoforms is regulated in individual cells and tissues, and the question what induces and regulates the expression of a particular mAC isoform in a cell certainly needs further study. For a long time, it is known that several mAC isoforms can be simultaneously expressed in a single cell resulting in different mAC isozyme ratios. The physiological mechanisms responsible for differential cellular expression patterns are unknown. Is the isozyme ratio in a cell stable over its lifetime or does it vary with development and aging? Distinct differences in cellular localization have been proposed and the concept of cAMP signaling compartmentation has been discussed in the past (Crossthwaite et al., 2005; Piggott et al., 2008; Ostrom et al., 2012; Scott et al., 2013; Cooper and Tabbasum, 2014; Johnstone et al., 2018). Another largely unanswered question is whether different mAC isozymes in a cell may be subject to distinct *direct* regulatory inputs, in addition to the well-established indirect activation *via* the GPCR axis. These findings and the number of unsolved questions suggests to consider that all mAC domains, cytosolic N- and C-termini, membrane anchors, catalytic domains, and the C1b linker regions which physically connect both halves of the protein, and possibly the C-termini are functionally indispensable, have distinct physiological roles and are required for regulating second messenger biosynthesis.

**FIGURE 3:**
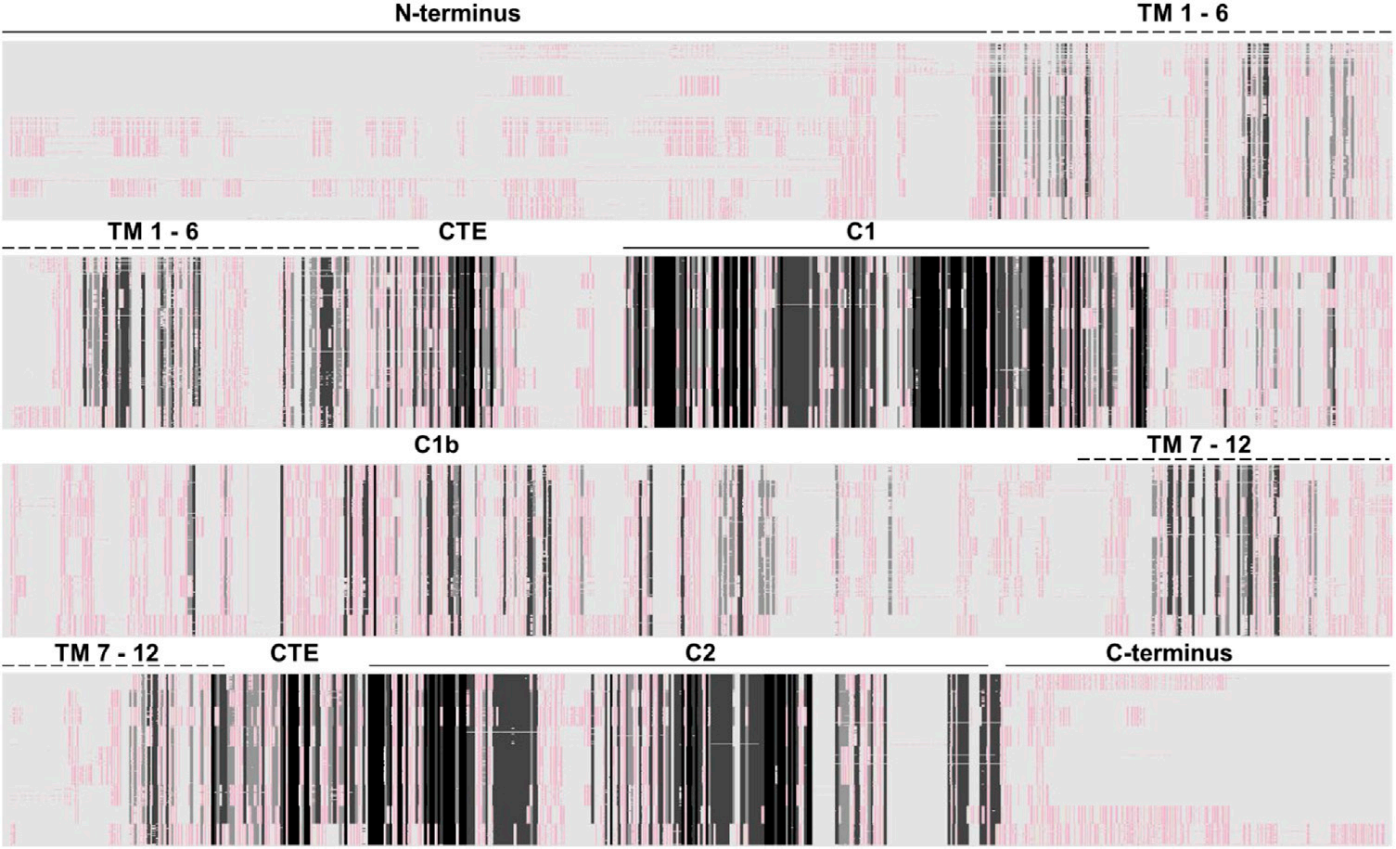
Alignment of 258 class III adenylyl cyclases about equally comprised of all nine isoforms (AC1 26 sequences; AC2, 25; AC3, 29; AC4, 30; AC5, 28; AC6, 30; AC7, 33; AC8, 28; AC9, 29). The approximate domain borders are indicated above. Clearly, the catalytic domains are cognizable as dark-shaded sections. All other domains, i.e., cytosolic N-and C-termini, the membrane domains and the cytosolic C1b linker are not conserved. The Clustal W alignment was adapted and shaded using the programme GeneDoc (http://nrbsc.org/gfx/genedoc/). Shading: black, invariant; dark grey, highly conserved; light grey, slightly conserved; whitish/reddish: fully diverged. A list of all mACs used for the alignment is attached at the end.

mACs are about similar in size, i.e., 1,065 (AC isoform 4)–1,353 amino acids long (AC isoform 9). A sequence alignment of 258 eukaryotic class III mAC isoforms shows that exclusively the two catalytic domains are conserved (Figure 3). All other domains are highly diverged. This is not surprising as mutations in the catalytic domains would have impaired enzyme activity and thus made the protein useless. In contrast, mutational events in associated domains probably resulted in gain of additional functional features [see Figure 1 and (Schultz and Natarajan, 2013)]. Generally, the cytosolic N-termini which precede the first 6TM anchor domain differ with respect to length and sequence. Similarly, the subsequent first hexahelical membrane domain (TM1) is poorly conserved among isoforms beyond the general fact that the hydrophobic amino acids Ala, Val, Leu, Ile predominate in transmembrane helices. The extra- and intracellular stretches between the membranous α-helices are rather short (Beltz et al., 2016). The catalytic C1 domain is connected to TM1 by a linker of about 80 aa containing a conserved stretch of 19 amino acids which constitutes a cyclase-transducing-element, abbreviated CTE, by others termed helical domain (Ziegler et al., 2017; Qi et al., 2019). In all nine human isoforms it is positionally conserved with respect to the start of the first catalytic domain, C1. CTE_1 has an invariant center sequence of SxL/MP [(Ziegler et al., 2017) and see below]. The C1 domain is sequence and length conserved, and this extends to its bacterial progenitor isoforms [(Bassler et al., 2018), see below].

What follows is an extended cytosolic domain, termed C1b, which in mACs 1-8 is approximately 145 aa–174 aa long, in mAC9 it is 205 aa long. C1b is not conserved between isoforms. Upon C1b follows the second hexahelical membrane domain (TM2) which is diverged. The linker between the exit from α-helix 12 of TM2 to the second catalytic domain; C2 is shorter compared to the similarly positioned linker in the first half of the protein. It carries a second, conserved sequence of 19 aa, CTE_2, with an almost invariant center, NxLP, significantly deviating from the corresponding CTE_1 (Ziegler et al., 2017). The second catalytic domain is conserved. The C-terminal regions (7 to almost 100 aa) are diverged. Similar comparisons have been made in the past and have been presented in various alignment formats.

## Examination of subdomain conservation in various categories of eukaryotic mAC isoforms

With the increasing number of fully sequenced vertebrate genomes comprehensive alignments allow valid predictions concerning potential mAC domain functions. Below I present exemplary samples of sequence comparisons of eukaryotic mAC domains using isoforms 1 to 9. In these alignments I span a huge evolutionary distance in each group with isoform sample sequences from the “living fossils” coelacanth and the elephant shark, birds, e.g., chicken, up to humans. The selected domain borders used for comparisons are uniform for all isoforms, however, due to inherent ambiguities should be considered as approximate borders. The presentation proceeds from N- to C-terminal.

### The N-termini are isoform-specifically conserved

**FIGURE 4:**
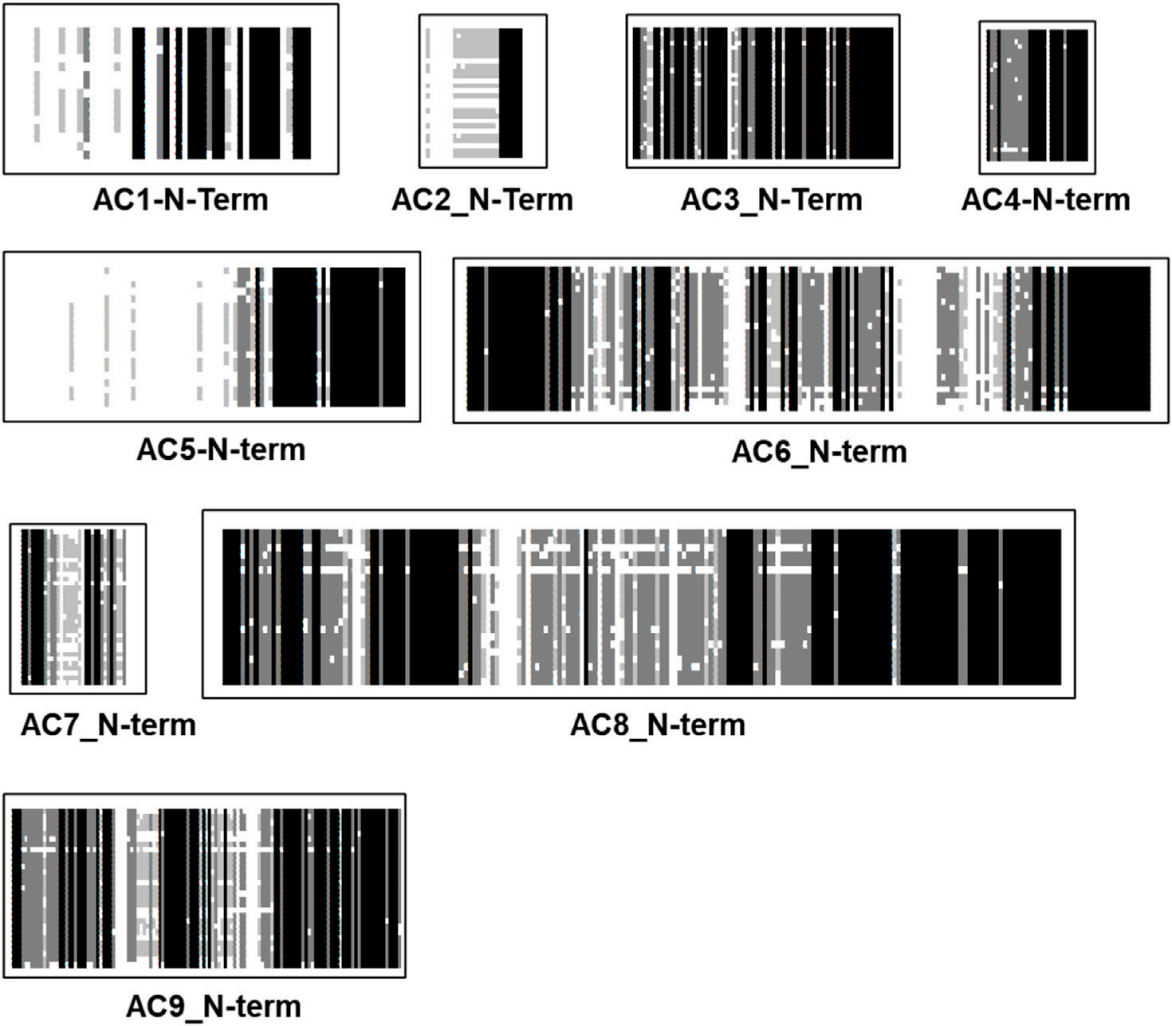
Alignment of N-termini of the nine mammalian mAC isoforms. mAC1: 19 isoforms were used, the N-terminus is 50 aa–60 aa long; mAC2: 27 isoforms; N-terminus rather variable between 6 aaand 50 aa. mAC3: 29 isoforms, length (77 aa) and sequence conserved. mAC4: 28 isoforms, length (28) and sequence conserved. mAC5: 28 isoforms of variable length (50 aa–240 aa); mAC6: 29 isoforms with an invariant 148 aa long, highly conserved N-terminus; mAC7: 34 isoforms, invariantly of 33 well conserved aa’s; mAC8: 29 isoforms, highly conserved length of 180 aa. mAC9: 34 isoforms, uniform length of 120 highly conserved aa. Note the invariant region prior to TM1 in all isoforms except AC7. Shading: black, invariant; dark grey, conserved; light grey, slightly conserved; white: disparate.

The N-termini of mACs are cytosolic. Potential bacterial progenitors have N-termini of variable length; sequence similarities are currently unknown. The biochemical and physiological functions of the N-termini of eukaryotic mACs have not been systematically investigated. An alignment of 258 N-termini (isoforms 1 through 9) shows no sequence conservation (see Figure 3). The N-termini vary in length from 6 aa to 240 aa. When examining N-termini of individual isoforms the situation changes profoundly (Figure 4). Most notably, the N-termini are isoform-specifically conserved for almost 0.5 billion years of evolution. Of note, all N-termini appear to have a short, invariant region ahead of the first α-helix of TM1. The length of the N-termini of mACs 3, 4, 6, 7, 8, and 9 is almost invariant (Figure 4). This suggest an indispensable physiological function which is currently unknown. Further to this point the isoform-singularity of the N-termini indicate that physiological functions of the N-termini vary with respect to isoform category. Presently, we can only speculate about potential functionalities, e.g., regulation of transcription, protein folding, targeted membrane insertion as reported for AC5 (Crossthwaite et al., 2005), cellular localization, and interactions with distinct regions of the catalytic dimer, possibly the C1b linker region or with other cellular proteins, as shown for the mAC2 and the A-kinase-anchoring protein Yotiao (Piggott et al., 2008).

### The membrane anchors are fully diverged, yet isoform-specifically conserved

The AC membrane anchor consists of two separate hexahelical domains which were predicted at the time the first mAC sequence was reported (Krupinski et al., 1989). In 2019 this prediction was experimentally verified by the cryo-EM structure of an mAC9 holoenzyme (Qi et al., 2019). Structure predictions by AlphaFold uniformly indicate two intertwined hexahelical membrane domains forming an aggregated structural entity. Initially, the membrane anchors were suggested to possess a function as ion channel or transporter, properties which subsequently could not be experimentally demonstrated (Krupinski et al., 1989). Another physiological function of the membrane anchors beyond membrane-anchoring was never outright dismissed, yet it appears difficult to experimentally probe such a possibility (Schultz and Natarajan, 2013; Beltz et al., 2016).

The two hexahelical membrane domains show little conservation beyond the usual predominance of hydrophobic amino acids. The eukaryotic TM1 and TM2 membrane domains are dissimilar to their bacterial congeners (Beltz et al., 2016). After the primordial gene-duplication/fusion event which involved a 6 TM bacterial AC progenitor mutational expansion of all domains except the catalytic domains progressed. Aligning TM1 and TM2 domains from 258 mACs isoforms 1 through 9 shows no sequence conservation (Figure 3). This demonstrates that in a time span of about 1 billion years of evolution the TM domains have isoform-specifically diverged beyond recognition from the supposed primordial bacterial fusion product with two identical membrane domains. Further, the TM1 and TM2 domains of every individual isoform when aligned against each other according to the α-helix predictions possess no sequence similarity.

**FIGURE 5:**
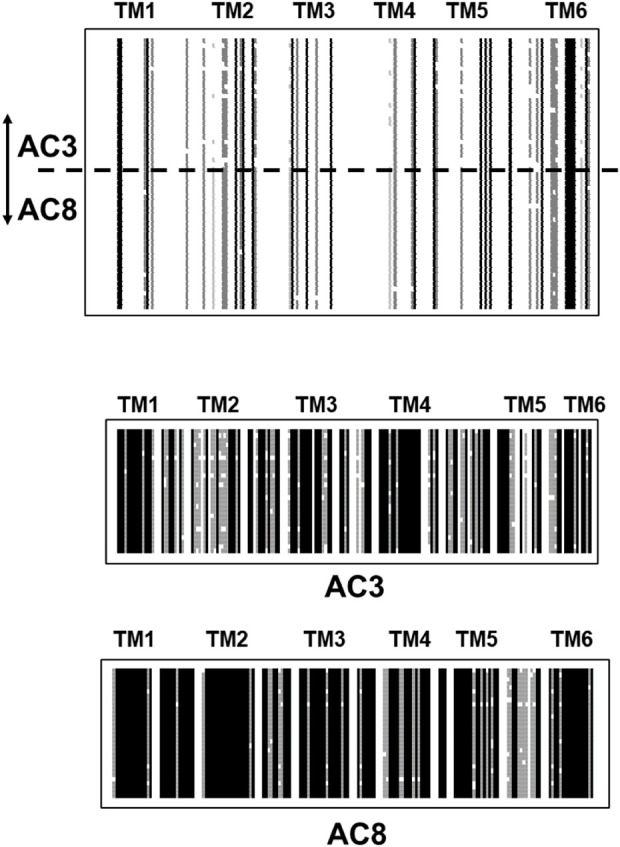
Top: Alignment of the first hexahelical membrane domains of mAC isoforms 3 (28 isoforms) and 8 (34 isoforms). The joint alignment shows scant conservation between the domains from mAC3 and 8. Single α-helices are indicated above the sequences. Below: alignments of the TM1 domains from mAC3 and mAC8. Note the high degree of conservation across a large variety of species. Shading: black, invariant; dark grey, conserved; light grey, slightly conserved; white: disparate. (Sequences used: AC3: Callithrix jacchus; alligator; antelope; ceratotherium simum simum; brown bat; chicken; chinchilla; desert mouse; dog; falcon; Mustela putorius furo; frog; goat; guinea pig; hog; horse; human; macaque; mouse; Heterocephalus; orca; coelacanth; rat; manatee; sheep; turtle; catfish. AC8: myotis brandtii; Bos taurus; camel; cat; chicken; dog; Mustela putorius furo; frog; gibbon; guinea pig; hog; human; hedgehog; lemur; macaque; monkey; mouse; Heterocephalus; rhino; orca; baboon; Ochotona princeps; pigeon; rat; manatee; sheep; Melopsittacus undulatus; squirrel; vicuna; walrus).

Strikingly, different pictures emerges when TM1 or TM2 domains of individual isoforms from creatures of vastly different evolutionary positions are aligned. The results unequivocally demonstrate an almost complete sequence conservation for about 500 million years, confirming again the fact that most of mAC evolution stopped at around this point in time. As an example, I present the alignment of TM1 of mAC3 (28 isoforms) and mAC8 (34 isoforms). mAC isoforms 3 and 8 are usually grouped together with mAC1 in one category because these isoforms are Ca^2+^-stimulated (other categories comprise Gβγ-stimulated mAC2, 4, 7; Giα/Ca^2+^-inhibited AC5 and 6, and supposedly forskolin-insensitive mAC9 (Sadana and Dessauer, 2009)). An alignment of the TM1 domains from mAC3 and 8 illustrates how different they are from each other (Figure 5 top). Of note, the last α-helix of this membrane domain shows an invariant stretch of 4 aa (LY/FMC) and an exactly spaced Gly. However, when TM1 domains from either mAC3 or mAC8 alone are aligned the unique identity is evident (Figure 5, middle and bottom).

I have systematically carried out such comparative alignments using all nine eukaryotic mAC isoforms with identical results: all TM1 membrane regions are highly conserved in an isoform specific manner across all species, yet are highly diverged when aligned pairwise in differing combinations or all together (see Figure 3). The same situation prevails when aligning the TM2 domains, i.e., in all animal species a high degree of sequence conservation within one isoform, yet no significant similarities when aligning TM2 domains from different mAC isoforms.

These sequence comparisons further confirm our earlier cluster analyses for mAC transmembrane domains. In these studies, the TM1 and TM2 domains clearly segregated according to isoform and the differences between TM1 and TM2 domains were resolved (Beltz et al., 2016). Previously, we had demonstrated, that it is impossible to generate chimeric mammalian mACs in which the membrane domains from different AC isoforms where combined (Seebacher et al., 2001). Thus, one can confidently conclude that TM1 and TM2 domains are not only highly isoform specific but share a pair-wise evolution, i.e., they are preserved as a functional pair for >450 million years. Clearly TM1 and TM2 domains evolved jointly to interact with each other. These observations indicate that the function of the dodeca-helical membrane domain of eukaryotic ACs is functionally not sufficiently described when attributing exclusively a simple anchoring function. We have proposed a receptor function for yet unknown ligands which directly modulate the extent of Gsα activation (Beltz et al., 2016; Ziegler et al., 2017; Seth et al., 2020).

### The conserved cyclase-transducing-elements

**FIGURE 6:**
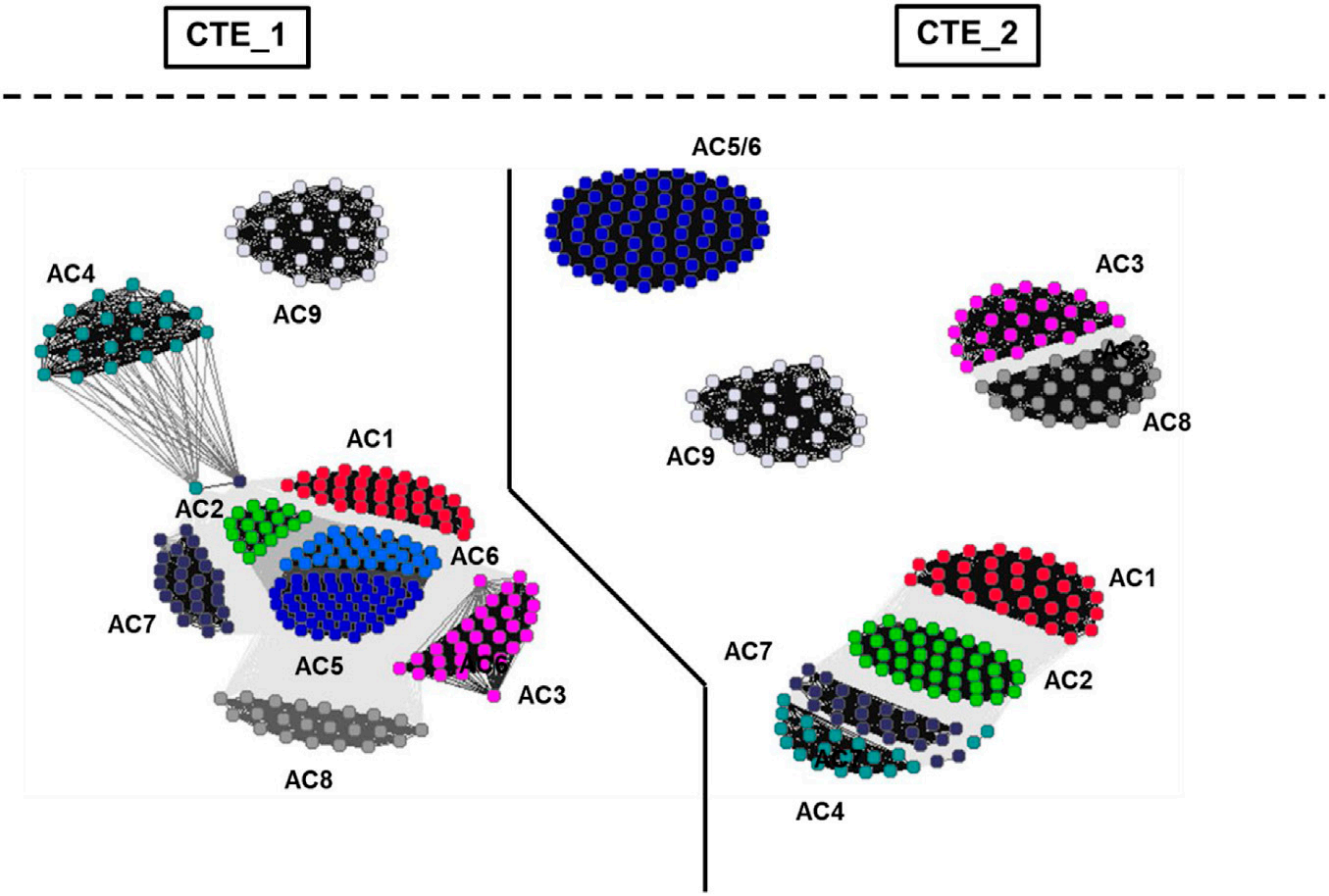
Cluster map of cyclase-transducing-elements (CTEs). For preparation of the figure, the data set from Ziegler et al. (2017) was used. The bacterial sequences were removed and vertebrate class IIIa CTEs were analyzed using CLANS (Frickey and Lupas, 2004). Each dot represents a single sequence. The CTE_1 and CTE_2 sectors are separated by a solid line. Cluster labeling indicates the mAC isoform. The segregation shows that CTEs from class IIIa ACs are highly specific for their C1- and C2-domain origins as well as for AC isoforms.

Evidently, this view is considerably bolstered by an analysis of the 19 aa long Cyclase-Transducing-Elements [CTE, (Vercellino et al., 2017; Ziegler et al., 2017)]. The CTEs are positioned between the membrane exit of TM1 or TM2, respectively, and the start of the subsequent catalytic regions, C1 or C2. CTE_1 and CTE_2 sequences differ. In a chimeric construct consisting of the hexahelical quorum sensing receptor LqsS from *Legionella pneumophila* and the mycobacterial AC Rv1625c it was demonstrated that the mycobacterial CTE is indispensable for signal transmission (Ziegler et al., 2017). A high resolution cluster analysis of the CTEs of eukaryotic mAC isoforms unequivocally demonstrated that they are position - and isoform-specifically conserved during evolution (Figure 6). This is almost unequivocal evidence that these CTEs must have an essential role in transmembrane signaling. Obviously, the catalytic domains are subjects of regulation by membrane signals and the CTEs are crucially positioned at the intersection (Ziegler et al., 2017).

### The catalytic domains C1 and C2

**FIGURE 7:**
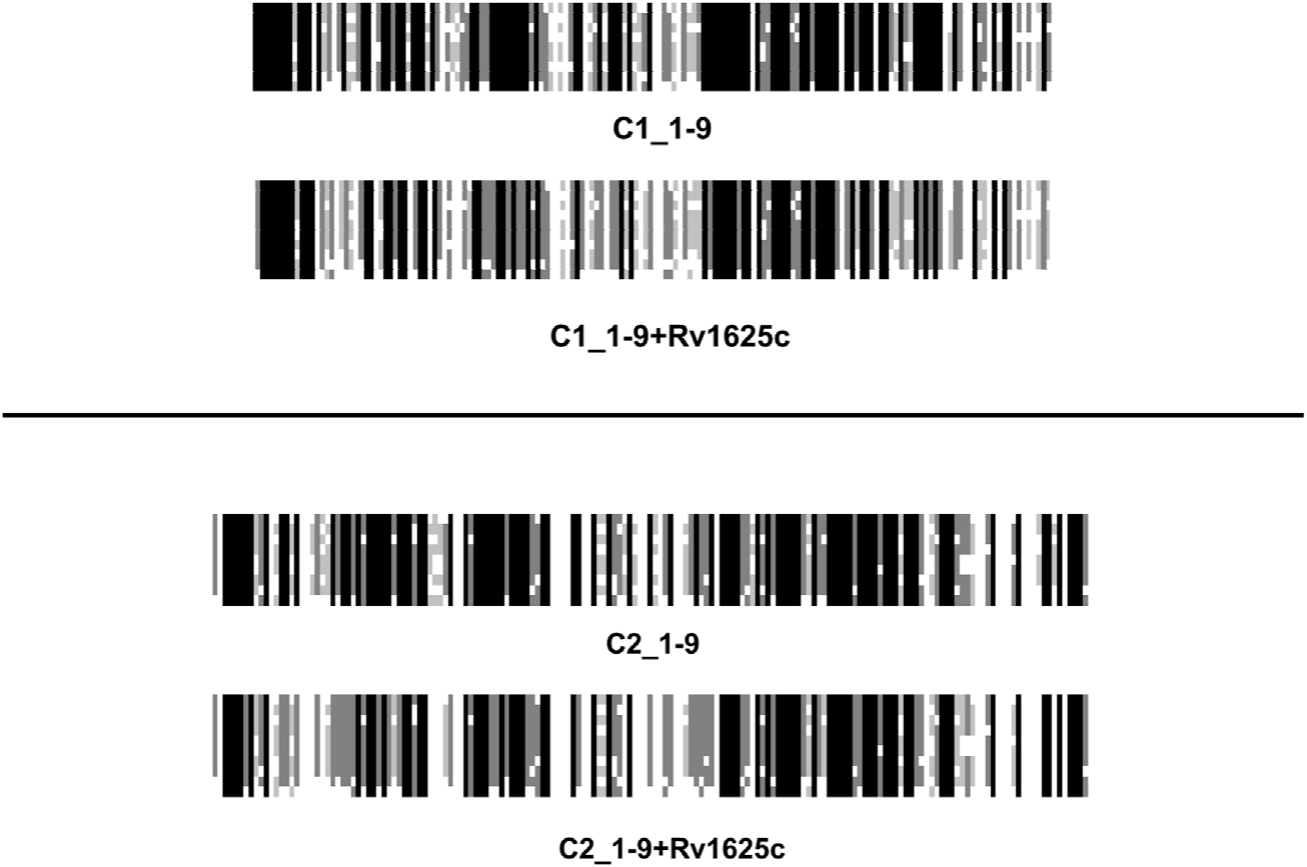
An alignment of the C1 and C2 catalytic domains from human adenylyl cyclases isoforms 1 through 9 ± the catalytic domain from the mycobacterial adenylyl cyclase Rv1625c.

The vertebrate mACs are termed “pseudoheterodimers” which denotes the identical domain compositions and sequences. That the AC signaling system evolved from a bacterial progenitor is most clearly shown by aligning the nine C1 or C2 catalytic domains ± the single catalytic domain from the mycobacterial AC Rv1625c, a potential prognitor (Figure 7). Due to the evolutionary history from cyanobacteria to mammals it is unsurprising that to conserve enzymatic functionality the catalytic domains of class III ACs display pronounced sequence similarities (Linder and Schultz, 2003). In the physically linked vertebrate “AC-dimers” the two catalytic domains C1 and C2 diverged only slightly. The catalytic amino acids, positionally conserved, are distributed between the C1 and C2 domains (Tesmer and Sprang, 1998). Neither C1 nor C2 by itself has catalytic activity. Extensive evolutionary mutations in the catalytic domains probably would have compromised activity and abolished system signaling capability. The major evolutionary gain in eukaryotic mACs then appears to be the acquisition regulation by G-proteins. Over the years we have been unable to unlock the molecular determinants which finally enable G-protein activation. We generated > 100 purposeful mutations in the mycobacterial mAC Rv1625c without attaining G-protein sensitivity (unpublished). The available structures of eukaryotic mACs obviously do not yet give a picture detailed enough to show exactly how Gsα-binds to and initiates the conformational changes leading to activation of the catalytic heterodimer (Tesmer et al., 1997; Dessauer et al., 1998; Tesmer and Sprang, 1998; Tesmer et al., 1999; Qi et al., 2019).

### The C1b domain

**FIGURE 8:**
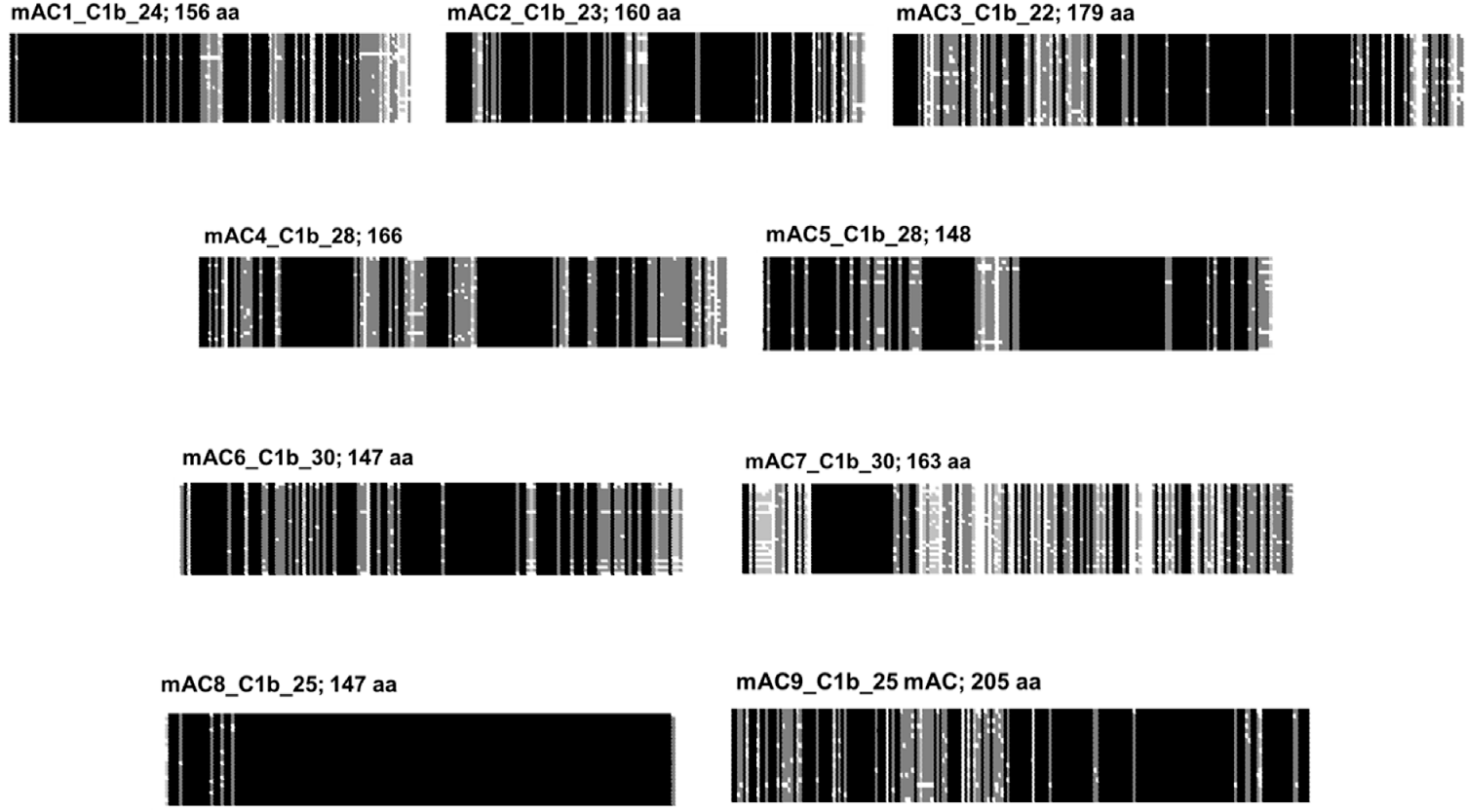
Alignment of the C1b subdomains of the mammalian mAC isoforms. The number of isozymes used for each alignment and the respective C-terminal lengths are indicated above. Shading: black, invariant; dark grey, conserved; light grey, slightly conserved; white: disparate.

C1b connects the halves of the pseudoheterodimer. Formally, one may assume that in the fusion event C1b originated from the C-terminus of the first half and the N-terminus of the second half. Resemblances to such partial sequences have not been identified, probably due to mutational adaptations during evolution. Combined alignments of C1b regions from 258 eukaryotic mAC isoforms show no similarity (see Figure 3). The conservation among any category of isoforms, however, is very high (Figure 8). This should be taken as an indication of an evolutionary functionalization of the C1b region fitting each mAC isoform with a peculiar regulatory potential. In fact, in the past the C1b region has been studied as a calmodulin-binding region in mAC isoforms, target of phosphorylation and of Ca^2+^-binding modulating activity.

### The C-terminal domains

**FIGURE 9:**
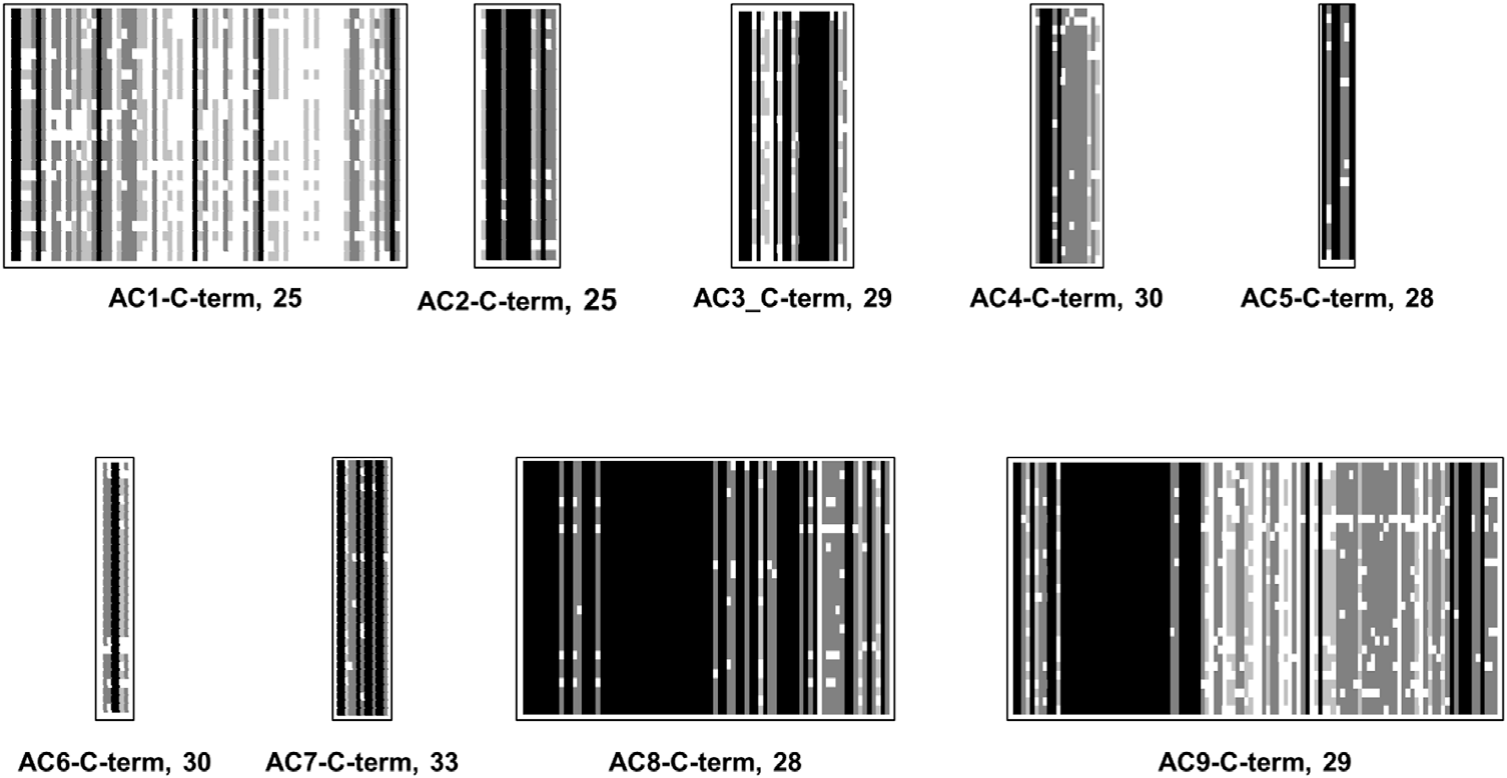
Alignment of C-termini of mammalian mAC isoforms. For each mAC isoform the number of analyzed proteins is indicated. mAC1: C-terminus is 65 aa–75 aa long. mAC2: C-terminus is uniformly 25 aa long. mAC3: C-terminus is 25 aa long. mAC4: length is 15 aa. AC5: length invariably 7 aa. mAC6: length 6 aa–7 aa. mAC7: 13 aa long; mAC8: highly conserved with a length of 81 aa. mAC9: uniform length of 110/111 aa’s. Shading: black, invariant; dark grey, conserved; light grey, slightly conserved; white: disparate.

Formally, the mAC C-terminal corresponds to the C1b region of the monomeric progenitor. Therefore, it is often termed C2b. Compared with C1b the sequences (148 aa–205 aa long) the C2b are shorter (25 aa–110 aa). No similarity is detected. The C-termini in general are diverged when compared as a set of 258 mACs (Figure 3), yet as with the other subdomains of mACs are highly conserved among isoforms (Figure 9). This would afford them distinct roles in mAC regulation. The general diversity and isoform conformity is indicative that no uniform functionality may be expected but functions tailored by evolution for each isoform.

## Discussion

The establishment of conserved sequence patterns of the nine eukaryotic mACs is necessarily a reduction which simplifies the complexity of mAC regulation. Yet even as a superficial sequence analysis it proves beyond a reasonable doubt that the system evolved up to a point around 0.5 billion years ago at which it reached a final state of functionality and, concomitantly, importance in regulating essential life functions. Obviously, this final evolutionary status of eukaryotic mAC isoforms was not visibly affected by subsequent events of whole-genome duplication in teleost’s about 350 to 216 million years ago (Glasauer and Neuhauss, 2014; Davesne et al., 2021), or the genome duplication in salmonids around 95 million years ago (Robertson et al., 2017). So, a comprehensive comparative analysis of protein sequences of mAC isoforms is presented. As such, this does not permit specific predictions concerning regulatory modalities connected with a specific subdomain. However, it should be helpful to focus future questions and experimental approaches systematically for each isoform on rather distinct domains of a mAC isoform. The established patterns of mAC regulation, as reported in the past (Sunahara et al., 1996; Sadana and Dessauer, 2009) should now be centered on individual isoforms and particular subdomains thereof. Each domain, i.e., N-termini, TM1 and TM2, C1b and C-termini appear to be fine-tuned to a specific isoform. It appears likely that patterns of regulatory inputs presently attributed to several isoforms are only an approximation.

In fact, over the years essentially all subdomains except for the TM1 and TM2 membrane anchors were implicated in one way or the other in regulatory mechanisms. The N-terminus of mAC isoform 6 was implicated in Gαi inhibition (Kao et al., 2004). The N-terminus of mAC8 was reported to bind protein phosphatase 2A (Crossthwaite et al., 2006; Simpson et al., 2006). The C1b region jointly with the N-terminus of mAC7 was implicated in the regulation by the G_13_ pathway (Jiang et al., 2013). The C-terminus of mAC2 has been implicated in regulation by phosphorylation (Levin and Reed, 1995; Böl et al., 1997a; Böl et al., 1997b; Pálvölgyi et al., 2018) and in mAC9 C2b region is implicated in autoinhibition (Vercellino et al., 2017; Qi et al., 2019; Qi et al., 2022). In mAC8 C2b has been reported as autoinhibitory and to contribute to the stimulation of the enzyme with Ca^2+^/calmodulin (Macdougall et al., 2009). An excellent example of the regulatory partition of the mAC subdomains may be the multiple mutations of mAC5 identified in humans. Thirteen point mutations have been identified. Affected are the N-terminus, the C1 domain, C1b domain, α-helix 7 of TM2, C2, and the C-terminus. Each mutation results in a distinct clinical pattern of hyperkinetic disorders (Ferrini et al., 2021).

A notable property of vertebrate mAC isozymes are the succinct differences of their 6TM membrane anchors. We can assume that after the early evolutionary dimerization event considerable diversification of the 6TM subdomains occurred. This fact may be interpreted in two ways: 1) the membrane domains may simply anchor the ACs into the cellular membrane and are otherwise physiologically inconsequential. The mutational diversifications may be fortuitous and of no further functional meaning. From this follows that one ascribes regulation of AC activity solely to the cytosolic portions of the proteins. 2) the succinct differences in membrane domains may be and, in the opinion of this author, should be taken as a sign of distinct regulatory properties affecting transmembrane signaling. One must ask why do nine vertebrate AC isoforms exist when these proteins anyway are uniformly activated by cytosolic Gsα, released upon GPCR activation and by forskolin, a non-physiologic activator of mAC isoforms (Seth et al., 2020)? To this author, signalling compartmentation appears a rather meager argument for disputing a regulatory functionality of the diverged membrane anchor. To plausibly explain how cAMP levels are regulated in mammals it will be necessary to incorporate a major and highly specific functionality of the membrane anchors. Needless to state that the consequences for physiology, pharmacology and the potential for development of therapeutics is enormous. With no chemically identified ligand the acceptance of a receptor hypothesis is, however, currently depending on whether one is willing to accept bioinformatic data as solid evidence and guidance for further explorations.

## Data Availability

The datasets presented in this study can be found in online repositories. The names of the repository/repositories and accession number(s) can be found in the article/Supplementary Material.
