# Supplementary material for: The evolutionary conservation of eukaryotic membrane-bound adenylyl cyclase isoforms
Source: Front Pharmacol. 2022 Sep 27;13:1009797. doi: 10.3389/fphar.2022.1009797 (PMC9552081; doi:10.3389/fphar.2022.1009797)
Supplement: Supplementary file 1 [file DataSheet1.pdf]

Supplementary material

List of eukaryotic adenylyl cyclases used in this study

AC1_Anas platyrhynchos	XP_005014606.1
AC1_Bos taurus	NP_776654.1
AC1_Canis lupus familiaris	XP_849081.3
AC1_Capra hircus	SXP_005679371.1
AC1_Cavia porcellus	XP_005001973.1
AC1_Ceratotherium simum simum	XP_004438438.1
AC1_Danio rerio	NP_001161822.1
AC1_Dasypus novemcinctus	XP_004454442.1
AC1_Ficedula albicollis	XP_005041477.1
AC1_Gallus gallus	XP_418883.4
AC1_Geospiza fortis	XP_005427323.1
AC1_Heterocephalus glaber	XP_004840600.1
AC1_Homo sapiens	NP_066939.1
AC1_Ictidomys tridecemlineatus	XP_005319314.1
AC1_Pseudopodoces humilis	XP_005525280.1
AC1_Macaca fascicularis	XP_005551359.1
AC1_Melopsittacus undulatus	XP_005148091.1
AC1_Mius musculus	O88444.2
AC1_Orca Orcinus orca	XP_004283063.1
AC1_Pan troglodytes	XP_519081.3
AC1_Pelodiscus sinensis	XP_006133339.1
AC1_Rattus norvegicus	XP_223616
AC1_Sarcophilus harrisii	XP_003762599.1
AC1_Xenopus	XP_002933461.1

AC2_Alligator mississippiensis	XP_006274555.1
AC2_Anas platyrhynchos	XP_005024930.1
AC2_Bos taurus	XP_587884.4
AC2_Callithrix jacchus	XP_002745178.1
AC2_Camelus ferus	XP_006195314.1
AC2_Canis lupus familiaris	XP_535798.3
AC2_Ceratotherium simum simum	XP_004440120.1

AC2_Columba livia	XP_005508897.1
AC2_Crassostrea gigas	EKC42885.1
AC2_Cricetulus griseus	EGW02068.1
AC2_Ficedula albicollis	XP_005041747.1
AC2_Geospiza fortis	XP_005427983.1
AC2_Homo sapiens	Q08462.5 GI:118572617
AC2_Ictalurus punctatus	AHH38946.1
AC2_Jaculus jaculus	XP_004670691.1
AC2_Latimeria chalumnae	XP_006007585.1
AC2_Macaca mulatta	NP_001252581.1
AC2_Monodelphis domestica	XP_001363692.1
AC2_Mus musculus	Q80TL1.2 GI:56748753
AC2_Myotis brandtii	EPQ02509.1
AC2_Ornithorhynchus anatinus	XP_001519046.2
AC2_Rabbit	XP_002721866.1
AC2_Rattus norwegicus	P26769.1 GI:117786
AC2_Trichechus manatus latirostris	XP_004380328.1

AC3_Alligator mississippiensis	XP_006258915.1
AC3_Bos taurus	NP_001193013.1
AC3_ Pantholops hodgsonii	XP_005978958.1
AC3_Callithrix jacchus	JAB47756.1
AC3_Canis lupus	XP_540108.3
AC3_Capra hircus	XP_005687005.1
AC3_Cavia porcellus	XP_003473206.1
AC3_Ceratotherium simum simum	XP_004418333.1
AC3_Chinchilla lanigera	XP_005400451.1
AC3_Chrysemys picta bellii	XP_005308548.1
AC3_Equus caballus	XP_001503112.2
AC3_Falco peregrinus	XP_005236727.1
AC3_Gallus gallus	XP_015140679.1
AC3_Heterocephalus glaber	XP_004839159.1
AC3_Homo sapiens	NP_004027.2
AC3_Ictalurus punctatus	AHI50404.1
AC3_Latimeria chalumnae	XP_006002174.1
AC3_Macaca mulatta	AFI36067.1

AC3_Microtus ochrogaster	XP_005360926.1
AC3_Mus musculus	NP_612178.2
AC3_Myotis davidii	XP_006767657.1
AC3_Myotis lucifugus	XP_006091753.1
AC3_Oreochromis niloticus	XP_005459452.1
AC3_Octodon degus	XP_004627610.1
AC3_Orcinus orca	XP_004268140.1
AC3_Ovis aries	XP_004005776.1
AC3_Rattus norvegicus	NP_570135.2
AC3_Sus scrofa	XP_003125378.1
AC3_Trichechus manatus latirostris	XP_004377624.1

AC4_Alligator mississippiensis	XP_006273291.1
AC4_Bos taurus	NP_001092678.1
AC4_Camelus ferus	XP_006172896.1
AC4_Canis lupus familiaris	XP_005623331.1
AC4_Capra hircus	XP_005685261.1
AC4_Cavia porcellus	XP_003474436.1
AC4_Ceratotherium simum simum	XP_004421230.1
AC4_Chelonia mydas	EMP26818.1
AC4_Cricetulus griseus	ERE89439.1
AC4_Echinops telfairi	XP_004698558.1
AC4_Equus caballus	XP_005603310.1
AC4_Heterocephalus glaber	EHB03385.1
AC4_Homo sapiens	NP_001185521.1
AC4_Jaculus jaculus	XP_004661893.1
AC4_Loxodonta Africana	XP_003421055.1
AC4_Mus musculus	NP_536683.1
AC4_Mustela putorius furo	XP_004755257.1
AC4_Nomascus leucogenys	XP_003260999.1
AC4_Ochotona princeps	XP_004584781.1
AC4_Octodon degus	XP_004647528.1
AC4_Odobenus rosmarus divergens	XP_004402087.1
AC4_Orcinus orca	XP_004283168.1
AC4_Ovis aries	XP_004010943.1
AC4_Pongo abelii	XP_002824659.1
AC4_Pteropus alecto	ELK11206.1
AC4_Rattus norvegicus	NP_062158.2

AC4_Saimiri boliviensis boliviensis	XP_003924311.1
AC4_Sus scrofa	XP_001927591.3
AC4_Trichechus manatus latirostris	XP_004390586.1
AC4_Tupaia chinensis	XP_006144671.1

AC5_Alligator mississippiensis	XP_006259479.1
AC5_Bos mutus	XP_005893490.1
AC5_Camelus ferus	XP_006189940.1
AC5_Canis lupus familiaris	NP_001161932.1
AC5_Ceratotherium simum simum	XP_004424848.1
AC5_Chinchilla lanigera	XP_005386584.1
AC5_Chrysemys picta bellii	XP_005279871.1
AC5_Danio rerio	NP_001165056.1
AC5_Dasypus novemcinctus	XP_004465968.1
AC5_Ficedula albicollis	XP_005049663.1
AC5_Gallus gallus	NP_989962.1
AC5_Heterocephalus glaber	XP_004873676.1
AC5_Homo sapiens	NP_899200.1
AC5_Ictidomys tridecemlineatus	XP_005338942.1
AC5_Mesocricetus auratus	XP_005071583.1
AC5_Microtus ochrogaster	XP_005344945.1
AC5_Nomascus leucogenys	XP_003275587.1
AC5_Macaca mulatta	AFE71085.1
AC5_Melopsittacus undulatus	XP_005153853.3
AC5_Mus musculus	NP_001012783.3
AC5_Ochotona princeps	XP_004577897.1
AC5_Orcinus orca	XP_004278874.1
AC5_Oryctolagus cuniculus	NP_001076097.1
AC5_Pantholops hodgsonii	XP_005982400.1
AC5_Rattus norvegicus	NP_072122.1
AC5_Trichechus manatus latirostris	XP_004373776.1
AC5_Tupaia chinensis	XP_006154659.1
AC5_Vicugna pacos	XP_006215832.1

AC6_Alligator sinensis	XP_006030803.1
AC6_Bos taurus	NP_001137349.1
AC6_Camelus ferus	XP_006177732.1
AC6_Canis lupus familiaris	NP_001182076.1
AC6_Capra hircus	XP_005680178.1
AC6_Cavia porcellus	XP_005006552.1
AC6_Chrysemys picta bellii	XP_005308461.1
AC6_Cricetulus griseus	EGW10407.1
AC6_Danio rerio	XP_002666536.2
AC6_Equus caballus	XP_005611190.1
AC6_Homo sapiens	NP_056085.1
AC6_Heterocephalus glaber	EHB00554.1
AC6_Ictalurus punctatus	AHH37241.1
AC6_Ictidomys tridecemlineatus	XP_005324943.1
AC6_Leptonychotes weddellii	XP_006729328.1
AC6_Macaca mulatta	EHH20682.1
AC6_Mus musculus	NP_031431.2
AC6_Myotis davidii	ELK36355.1
AC6_Orcinus orca	XP_004274438.1
AC6_Pantholops hodgsonii	XP_005981059.1
AC6_Pan troglodytes	XP_509033.3
AC6_Pongo abelii	XP_002823208.1
AC6_Pseudopodoces humilis	XP_005532101.1
AC6_Pteropus alecto	ELK08411.1
AC6_Sorex araneus	XP_004616974.1
AC6_Sus scrofa	XP_005655595.1
AC6_Rattus norvegicus	NP_001257714.1
AC6_Tupaia chinensis	ELV10887.1
AC6_Vicugna pacos	XP_006202980.1
AC6_Xenopus	XP_002935166.2

AC7_Alligator mississippiensis	XP_006273282.1
AC7_Anas platyrhynchos	EOA99812.1
AC7_Bos taurus	NP_776655.1
AC7_Callorhinchus milii	XP_007887633.1
AC7_Camelus ferus	XP_006183595.1
AC7_Cavia porcellus	XP_003477728.2
AC7_Ceratotherium simum simum	XP_004431649.1
AC7_Columba livia	XP_005504744.1

AC7_Cricetulus griseus	ERE77536.1
AC7_Danio rerio	NP_001159744.1
AC7_Felis catus	XP_003998076.1
AC7_Ficedula albicollis	XP_005052707.1
AC7_Gallus gallus	XP_414097.4
AC7_Geospiza fortis	XP_005426511.1
AC7_Gorilla gorilla gorilla	XP_004057679.1
AC7_Ictidomys tridecemlineatus	XP_005318224.1
AC7_Jaculus jaculus desert	XP_004664906.1
AC7_Heterocephalus glaber	XP_004904810.1
AC7_Homo sapiens	NP_001105.1
AC7_Loxodonta africana	XP_003416421.1
AC7_Melopsittacus undulatus	XP_005152446.1
AC7_Mus musculus	NP_001103226.1
AC7_Ochotona princeps	XP_004584171.1
AC7_Odobenus rosmarus divergens	XP_004408580.1
AC7_Orcinus orca	XP_004265110.1
AC7_Pelodiscus sinensis	XP_006121204.1
AC7_Pseudopodoces humilis	XP_005526497.1
AC7_Rattus norvegicus	NP_445848.1
AC7_Trichechus manatus latirostris	XP_004371655.1
AC7_Tupaia chinensis	XP_006154858.1
AC7_Vicugna pacos	XP_006207919.1
AC7_Zonotrichia albicollis	XP_005488910.1
AC8_Bos taurus	NP_001179770.1
AC8_Callithrix jacchus	JAB44699.1
AC8_Camelus ferus	XP_006189280.1
AC8_canis lupus familiaris	XP_005627997.1
AC8_Cavia porcellus	XP_003467396.1
AC8_Ceratotherium simum sinum	XP_004431120.1
AC8_Columba livia	XP_005510872.1
AC8_Danio rerio	NP_001137224.1
AC8_Felis catus	XP_004000169
AC8_Gallus gallus chicken	XP_418437.2
AC8_Heterocephalus glaber	XP_004837996.1
AC8_Homo sapiens	NP_001106.1
AC8_Ictidomys tridecemlineatus	XP_005316205.1
AC8_Macaca fascicularis	EHH64433.1
AC8_Melopsittacus undulatus	XP_005143885.1
AC8_Mus musculus	NP_033753.2

AC8_Mustela putorius furo	XP_004786573.1
AC8_Myotis brandtii	EPQ11792.1
AC8_Nomascus leucogenys	XP_003256260.1
AC8_Ochotona princeps	XP_004580789.1
AC8_Orcinus orca	XP_004265364.1
AC8_Otolemur garnettii	XP_003782388.1
AC8_Papio anubis	XP_003903213.1
AC8_Rattus norvegicus	NP_058838.1
AC8_Trichechus manatus latirostris	XP_004373065.1
AC8_Vicugna pacos	XP_006208083.1

AC9_Alligator mississippiensis	XP_006266933.1
AC9_Bos taurus	NP_001192846.1
AC9_Capra hircus	XP_005697982.1
AC9_Ceratotherium simum simum	XP_004438179.1
AC9_Columba livia	XP_005505680.1
AC9_Danio rerio	XP_003201145.1
AC9_Equus caballus	XP_001502298.1
AC9_Falco cherrug	XP_005436868.1
AC9_Felis catus	XP_003998961.1
AC9_Ficedula albicollis	XP_005054227.1
AC9_Gallus gallus	NP_989961.1
AC9_Geospiza fortis	XP_005425327.1
AC9_Gorilla gorilla gorilla	XP_004057148.1
AC9_Heterocephalus glaber	EHB15048.1
AC9_Ictalurus punctatus	AHH39076.1
AC9_Jaculus jaculus	XP_004652275.1
AC9_Homo sapiens	NP_001107.2
AC9_Latimeria chalumnae	XP_005990721.1
AC9_Melopsittacus undulatus	XP_005152541.1
AC9_Mesocricetus auratus	XP_005081721.1
AC9_Mustela putorius furo	XP_004797716.1
AC9_Myotis davidii	ELK34669.1
AC9_Mus musculus	NP_033754.
AC9_Ochotona princeps	XP_004586761.1
AC9_Odobenus rosmarus divergens	XP_004403278.1
AC9_Orcinus orca	XP_004270284.1
AC9_Pseudopodoces humilis	XP_005522877.1
AC9_Saimiri boliviensis boliviensis	XP_003928522.1

AC9_Sorex araneus	XP_004604427.1
AC9_Taeniopygia guttata	XP_002194895.1
AC9_Trichechus manatus latirostris	XP_004373265.1
AC9_Xenopus laevis	NP_001079302.1
